# Evaluating Surveillance Breast Imaging and Biopsy in Older Breast Cancer Survivors

**DOI:** 10.1155/2012/347646

**Published:** 2012-10-14

**Authors:** Tracy Onega, Julie Weiss, Roberta diFlorio, Todd MacKenzie, Martha Goodrich, Steven Poplack

**Affiliations:** ^1^Department of Community and Family Medicine, Geisel School of Medicine at Darmouth, Lebanon, NH 03756, USA; ^2^The Dartmouth Institute for Health Policy and Clinical Practice, Geisel School of Medicine at Darmouth, Lebanon, NH, USA; ^3^Norris Cotton Cancer Center, Dartmouth Hitchcock Medical Center, One Medical Center Drive, Lebanon, NH 03756, USA; ^4^Department of Radiology, Geisel School of Medicine at Darmouth, One Medical Center Drive, Lebanon, NH 03756, USA; ^5^Department of Medicine, Geisel School of Medicine at Darmouth, Lebanon, NH, USA

## Abstract

*Background*. Patterns of surveillance among breast cancer survivors are not well characterized and lack evidence-based practice guidelines, particularly for imaging modalities other than mammography. We characterized breast imaging and related biopsy longitudinally among breast cancer survivors in relation to women's characteristics. *Methods*. Using data from a state-wide (New Hampshire) breast cancer screening registry linked to Medicare claims, we examined use of mammography, ultrasound (US), magnetic resonance imaging (MRI), and biopsy among breast cancer survivors. We used generalized estimating equations (GEE) to model associations of breast surveillance with women's characteristics. *Results*. The proportion of women with mammography was high over the follow-up period (81.5% at 78 months), but use of US or MRI was much lower (8.0%—first follow-up window, 4.7% by 78 months). Biopsy use was consistent throughout surveillance periods (7.4%–9.4%). Surveillance was lower among older women and for those with a higher stage of diagnosis. Primary therapy was significantly associated with greater likelihood of breast surveillance. *Conclusions*. Breast cancer surveillance patterns for mammography, US, MRI, and related biopsy seem to be associated with age, stage, and treatment, but need a larger evidence-base for clinical recommendations.

## 1. Introduction

The National Cancer Institute (NCI) defines a cancer survivor as anyone who has been diagnosed with cancer, regardless of time since diagnosis, treatment status, or overall prognosis [1]. Because the overall mortality rates for breast cancer are relatively low while incidence is relatively high, there are estimated to be more than 2,000,000 women in the U.S. who are breast cancer survivors [2]. For these women, breast surveillance following completion of treatment is based on guidelines recommending annual surveillance mammography [3, 4], and on adoption of new technologies such as breast MRI [5]. Recommendations for annual mammographic surveillance do not stem from clinical trial evidence [6–9], but on evidence from observational studies and consensus panels. Consensus opinion holds that women with a personal history of breast cancer may benefit from early detection of subsequent breast cancers. Risk of new or recurrent breast cancer is increased among these women compared to women with no history of breast cancer. Survivors are at a 2 to 6 times greater risk of a new primary in the contralateral breast [10]. The overall risk of a recurrence or new primary breast cancer is estimated to be 5.4–6.6/1,000 woman-years [11].

Despite recommendations for annual mammography put forth by entities, such as the National Comprehensive Cancer Network, the American Cancer Society, and the American College of Radiology, breast surveillance has been shown to be low among women who are elderly, black, had late-stage disease, had mastectomy or breast conserving surgery (BCS) without radiation, did not see a physician, and had more comorbid illnesses [12–16]. Also, adherence to surveillance mammography diminishes over time, with one study showing a decline from 80% to 63% over five years for women undergoing annual mammography [13]. 

Guidelines do not provide evidence-based recommendations for how long annual mammography should be continued in women with a personal history of breast cancer—a particularly relevant issue for elderly breast cancer survivors whose life expectancies may not yield any potential benefit from early detection of breast cancer. Further, there is an absence of evidence or recommendations for surveillance use of other imaging modalities, such as ultrasound or breast MRI for surveillance, although reports demonstrate the use of these modalities for surveillance [17]. Guidelines from the American Cancer Society [18], which are endorsed by the European Society of Breast Imaging [19] and other groups, suggest MRI screening for women with a lifetime breast cancer risk ≥20% and *BRCA* gene mutation carriers based on evidence of value in these high-risk groups [20–24]. However, there is specific inclusion of personal history of breast cancer in these MRI guidelines. Also, without validated risk models for specific factors related to risk of a second breast cancer, it is difficult for physicians to risk-stratify patients for determining surveillance management, such as how frequently to screen over time, which imaging modalities to use, and how long to continue surveillance as women age.

There is recognition that surveillance patterns should be tailored to women's informed decisions based on the evidence that does exist for benefits and harms [6]. Still lacking, however, is a comprehensive understanding of how much and what kind of breast surveillance is occurring among women with a personal history of breast cancer.

This study provides a more complete characterization of surveillance patterns among women with a personal history of breast cancer than has been reported to date. As in other studies, we examined factors associated with mammography, but have included family history, which has not been previously reported. We also provided longer follow-up over which surveillance was assessed. Finally, we included all major breast imaging modalities—mammography, ultrasound, and MRI, as well as related breast biopsies in our characterization of surveillance patterns. 

## 2. Methods

### 2.1. Data Sources and Linkage

The New Hampshire Mammography Network (NHMN) registry has collected mammographic information since 1995 [25]. In 1999, the NHMN became part of a national breast cancer surveillance program, funded by the National Cancer Institute (NCI), known as the Breast Cancer Surveillance Consortium (BCSC) [26]. In 2008, NCI worked with Center for Medicare & Medicaid Services (CMS) to link BCSC data to the Medicare claims data. The BCSC-Medicare linkage provides a unique database that includes claims covering years 1998–2006.

From the 1998–2006 BCSC-Medicare linked file, the BCSC identified 4,242 NHMN women with breast cancer, their date of diagnosis and type of cancer. Using a unique BCSC ID variable, the 4,242 NHMN women and their NHMN data records were linked to the Medicare files. The Medicare Outpatient and Carrier files, were used to determine breast events including mammography, ultrasound, magnetic resonance imaging (MRI), and biopsy; comorbidities were calculated using the Medicare Outpatient, Carrier, and the review file (MEDPAR); the Medicare enrollment file, used to determine women's date of enrollment in Medicare; and NHMN data, used to determine family history of breast cancer and education, were utilized to characterize breast surveillance (image and biopsy) in relation to age at diagnosis, cancer type and stage, primary therapy, family history of breast cancer, comorbidities, and education.

### 2.2. Population

Using the Medicare enrollment file for the 4,242 women with a breast cancer, we selected 3,899 women who had “aged in”, that is, turned 65 sometime during 1998–2006. To ensure a woman had at least 18 months of follow-up and 6 months of Medicare data after a cancer diagnosis, she had to be eligible for Medicare 6 months before her date of diagnosis and her cancer diagnosed in the time period between June 1, 1998 and January 1, 2005; thereby, we removed 1,869 women who did not meet these conditions. Woman younger than 64 years at time of diagnosis (*N* = 484) and those with a bilateral mastectomy (*N* = 15) were removed. Since imaging in more advanced stages may represent continued management of metastatic disease, women with stage IV (*N* = 196) were removed. Women without at least one primary or specialist visit during an 18 month surveillance window were removed (*N* = 60), resulting in a total of 1,275 women eligible for this analysis. 

### 2.3. Surveillance Windows

**Figure 1:**
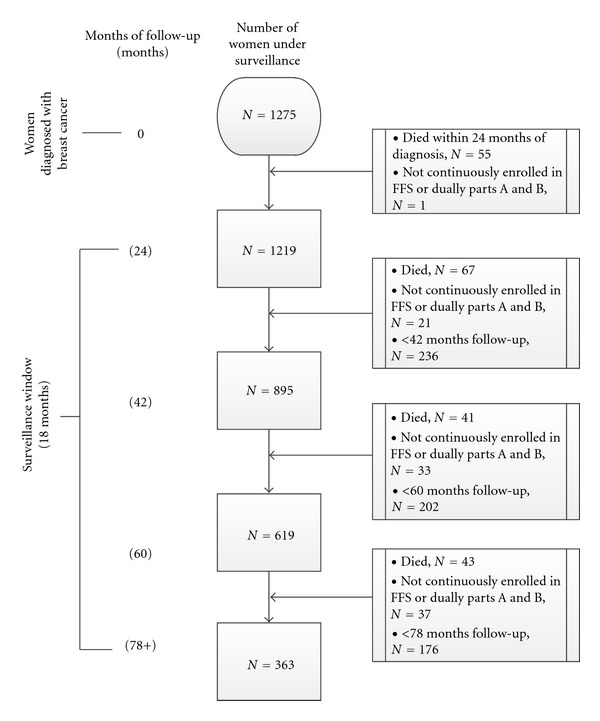
Surveillance windows.

We defined surveillance broadly as breast imaging occurring at least 6 months after a woman's breast cancer diagnosis. We included breast biopsies in order to characterize the totality of breast care women receive following primary treatment of breast cancer. The surveillance window began 6 months after a breast cancer diagnosis and continued through the end of the study period, with discrete surveillance windows categorized in increments of 18 months.  Figure 1 shows the specific inclusion criteria for each of the 18 month surveillance windows. Deceased women or women who were lost to follow up during each 18 month window were excluded. Women were only represented in a given window if they completed the time period. Since Medicare does not receive billing claims for physician services from either HMO plans or missing Part B, women participating in HMO plans or who were not continuously enrolled in Medicare dual Parts A and B throughout a particular window were removed. The first 18 month window included 1,219 of the 1,275 women. Of the 1,219 women, 895 women were followed for 42 months, 619 women were observed for 60 months and 363 had 78 months of continued surveillance (Figure 1 Derivation of Study Population and Surveillance Windows).

### 2.4. Outcomes

The primary endpoints were the occurrence of any breast imaging event of a mammogram, ultrasound, MRI, and related breast biopsies. Current procedural terminology codes (CPT), healthcare common procedure coding system (HCPCS), and international classification of disease, 9th edition codes (ICD-9) were used to identify mammograms (diagnostic, screening, any mammography), other breast imaging (ultrasound and MRI), and breast biopsies from Outpatient and Carrier Medicare files. Indicator variables were created for any mammogram, ultrasound or MRI breast event, or breast biopsy during each surveillance window. The sum of all breast events was calculated for each woman during each 18 month window.

### 2.5. Patient Characteristics

The date of diagnosis, type, and stage of breast cancer were provided in the BCSC-Medicare linked file. Age at diagnosis was calculated from date of diagnosis and the birthdate indicated in the Medicare file. Family history of breast cancer (indicated by first degree relatives of mother, daughter, or sister) and education were provided in the NHMN data.

Primary therapies of mastectomy, breast conserving surgery (BCS) with radiation, BCS without radiation, and unknown/other occurring within the first six months after diagnosis were calculated using breast procedure codes in the Outpatient and or Carrier files. A hierarchical assignment was used to designate primary therapies if more than one therapy was used during the six months. If a primary therapy was used that did not fall into the three categories of mastectomy, BCS with radiation, or BCS without radiation, an unknown or other category was appointed. 

A modified Charlson comorbidity index algorithm [27] reflecting the Deyo and Romano adaptations for classifying prognostic comorbidities with respect to breast cancer was used. The code uses diagnostic and surgery codes found in Medicare's Outpatient, Carrier, and MEDPAR files. We defined the number of primary care or specialty visits and the proportion of specialty care visits a woman had during a surveillance window using the healthcare financing administrative provider specialty codes (HCFASPEC) from the Carrier file. 

### 2.6. Analysis

For each surveillance window, utilization rates for each breast imaging (mammogram and combined ultrasound and MRI), biopsy, and breast events by the patient characteristics of age at diagnosis, stage of diagnosis, primary therapy, family history of breast cancer, comorbidities, and education were calculated. Additionally, overall utilization rates were computed for breast imaging, biopsy, and breast events per surveillance window. Our data is “unbalanced”; that is, not all of the women were in all 4 surveillance windows. In order to characterize the “population-average” or the mean response of breast events over all the windows by the women's characteristics, accounting for both repeated measures and correlated observations, we used generalized estimating equations (GEE) to estimate the incidence rate ratios for each of the outcomes [28]. Poisson models with a log link were implemented for both count data and binary outcomes. Risk ratios are more meaningful than odds ratios when the outcome is common, such is the case in our study for mammograms. GEEs were chosen to correct the standard errors (SE) by using robust SEs in the Poisson models for binary outcomes. Clustering on the woman ensured that the standard errors were not underestimated. We assumed a separate correlation for each pair of time points and therefore assigned an unstructured correlation matrix. Models were adjusted for all patient characteristics, including but not reported, the number of primary or specialty visits, proportion of specialty care visits, and diagnosis year. We report incidence rate ratios and 95% confidence intervals (95% CI). SAS 9.2 and StataSE 12 were used for analyses.

## 3. Results

Of the 1,219 women, most (59.3%) were less than 75 years of age, had stage I disease (55.4%), were treated with mastectomy (68.3%), had no family history of breast cancer (71.8%), and no comorbidities (65.7%) (Table 1).

Except for biopsy rates, the proportion of overall women with surveillance declined over the follow-up time for imaging. The proportion of women receiving mammography by 24 months was 89.3%, and decreased to 81.5% by 78 months of follow-up (Table 2). For ultrasound and MRI, the rate of use during the first surveillance window was 8.0%, but was 4.7% by the last window (Table 3). Biopsy was comparable across windows (range: 7.4%–9.4%, Table 4). 

Examining mammography surveillance patterns by age showed a greater decline over time with each successively older age group (Table 2). This pattern was not observed for advanced imaging modalities (US and/or MRI). Women in the 65–69 yr. age group had an 11.1% rate of advanced imaging from 6 to 24 months from diagnosis and 4.7% from 60 to78 months. Of women ages 75–79 and 80+, 6.2% and 5.4%,respectively, had US and/or MRI in the first surveillance window. By the last surveillance window, the proportions were similar (6.0% and 6.1%, Table 2). Biopsy use across all windows was somewhat high for 65–74 year olds (Table 3). Even in the 60–78 month window, biopsy rates were 10.3% for 65–69 yrs. and 13.8% for 70–74 yrs. 

In the first surveillance window, the unadjusted rates of mammography by stage at diagnosis were higher for DCIS (91.0%) and stage I (91.3%) than for stages IIA (85.7%), IIB (78.5%), and III (81.1%) (Table 2). For all stages except IIB, declines over time were noted. Similar trends were seen for US and MRI (Table 3). Higher stage demonstrated generally higher rates of biopsy, but no declines over time were noted (Table 4). 

Surveillance in relation to primary therapy types, showed crude rates of mammography (Table 2) and biopsy (Table 4) highest across all follow-up windows for women with BCS + radiation. In contrast, women with BCS alone showed higher rates of advanced imaging (Table 3). There were no clear trends in surveillance imaging or biopsy when examined crudely in relation to family history of breast cancer, comorbidities, and education. 

In multivariable poisson GEE models, we found increasing age to significantly lower the incidence of mammography, biopsy, and total breast events, particularly for the 80+ age group (Table 5). In general, higher stage at diagnosis was associated with lower incidence of surveillance. For example, the incidence rate ratio for total number of breast events for women with stage IIA compared to stage 1 was 0.85; 95% CI 0.79–0.92; for stage IIB versus I, 0.81; 95% CI 0.71–0.94, and for stage III versus I, 0.65; 95% CI 0.52–0.81. Primary therapy was significantly associated with surveillance. Women with BCS + radiation were more likely to have mammography compared to women with unilateral mastectomy (IRR = 1.09; 95% CI 1.06–1.12, mastectomy referent). For US and/or MRI, women with BCS alone were more likely to receive this advanced imaging surveillance (IRR = 1.68; 95% CI 1.06–2.66, mastectomy referent). For total breast events, BCS ± radiation was significantly associated with an increased incidence compared to unilateral mastectomy (Table 5). In our multivariable models, no significant associations were seen with the breast imaging surveillance measures and family history of breast cancer, comorbidity status, or education. 

## 4. Discussion

Using a state-based mammography registry linked to Medicare claims, we elucidated patterns of breast surveillance extending to at least 6.5 years following completion of primary treatment among women with breast cancer. A high proportion of women underwent surveillance mammography, although this proportion declined steadily over time. When adjusting for time since diagnosis, and important covariates, age but not comorbidity was significantly associated with a decreased use of mammography and breast biopsy. Primary therapy seemed to influence use of breast imaging; compared to mastectomy, BCS plus radiation was significantly associated with greater use of mammography, and BCS alone with advanced imaging modalities. Stage at diagnosis was also significant in surveillance patterns, with higher invasive stages less likely to receive surveillance than stage I. The intensity of surveillance, as measured by number of breast events, seems to decrease with age and stage at diagnosis, and increase for women who had BCS (with or without radiation) compared to unilateral mastectomy. Family history and comorbidities were not significantly related to surveillance. 

This study supports the findings of previous work showing a decline in surveillance mammography over time [12–16], but additionally demonstrates this effect over a longer time period than previously reported. Our findings suggest that the decline in mammography over time is more notable among the older Medicare population, which may reflect a decreasing likelihood of benefit from surveillance mammography as women age, since early detection of breast cancer is less likely to reduce mortality among those with diminished life expectancy. We also show a similar overall trend of decline for ultrasound and MRI, although without the same apparent effect of age. In fact, US and MRI occur at almost twice the rate for younger Medicare beneficiaries than older women early in the surveillance period, but was the same later. Unlike imaging, breast biopsy use showed no change over time. One possible interpretation of this result is that imaging tests may be more discretionary, but once an abnormal imaging test occurs, biopsy is the definitive test to diagnose a lesion. In examining factors associated with breast surveillance while adjusting for other variables, we are among the first to report on total number of imaging and/or biopsy events, which help to characterize intensity of breast care from the women's perspective. This measure helps our understanding of how the experience of surveillance may differ from screening in an older population. The number of breast events is lower for women over age 75 compared to 65–74. Whether this result is due to weighing of competing risks, use of general screening guidelines, or other reasons is unknown. The lower number of breast events as stage at diagnosis increases is difficult to interpret, but may represent bias in that women with early stage breast cancer may be a population more likely to be screened or to have benefited from screen detection, and therefore also more likely to undergo surveillance. In contrast women with later stage disease either may not have benefited from screening, that is, experienced false negative mammography, or be more likely to have their index cancer come to light clinically. 

Similarly unclear are the reasons for higher breast events among women with breast conserving surgery (BCS) (with or without radiation) compared to women with mastectomy. This finding may also reflect selection bias, as the need for less intensive surveillance maybe a reason for selecting mastectomy from the outset. Increased surveillance in women with BCS (with or without radiation) may be due to a perception that having more remaining breast tissue suggests a greater need for imaging or be due to a perception of insufficient treatment in women undergoing BCS only. Because there was no association between biopsy and primary therapy, the relation between number of breast events (biopsy plus imaging) and primary therapy is attributable only to imaging. Other studies have also reported a higher likelihood of mammography among women with BCS plus radiation, but not among those without radiation [12–16]. These studies have suggested possible reasons, such as access issues, socioeconomic factors, or communication deficits. Interestingly, our findings for US and/or MRI show significant increase for women with BCS alone (no radiation) compared to mastectomy, but no similar increase for women with BSC with radiation. This may reflect a bias that women selecting BCS only are concerned about radiation exposure. Nevertheless, although women with BCS without radiation may possibly be receiving less mammography than is recommended, they are receiving more advanced imaging than women with other primary treatments, and thereby may be undergoing surveillance. 

In prior studies of surveillance mammography, the influence of family history on use patterns has not been reported. Although family history of breast cancer is an important factor in breast cancer risk models, there are currently no accepted, validated risk models for subsequent breast tumor events (recurrence or new primary) among women with a personal history of breast cancer. Thus, without knowing the role family history plays in risk for these women, it is difficulty to weigh the importance of family history in breast surveillance decisions. Nevertheless, it is possible that women and/or providers *perceive* an increased risk and potential increased benefit from breast surveillance, and therefore use it more. However, our null finding in the association between family history and breast surveillance use suggests that this factor is not significant to women and/or providers in surveillance decisions. 

Unlike in some studies [12–14], we did not see any significant associations of breast surveillance imaging or biopsy with comorbidity status. Our comorbidity measure was based prior to breast cancer diagnosis, while others included a period after diagnosis. Further, we used an absolute count of comorbidities, rather than quantiles based on our study population. One would expect to see a decrease in breast imaging surveillance among Medicare-aged women with high-comorbidity burdens, since the likelihood of benefit from early detection is low. It is possible that the observed decrease in surveillance with age partly reflects increasing comorbidities occurring with advancing age that has not been captured by our methodology. 

This study is the first, to our knowledge, to report on breast surveillance use beyond just mammography. Although inclusion of US, MRI, and biopsy is a strength of this study, we acknowledge the limitation of using Medicare claims data to ascertain breast-related utilization in the surveillance periods. Medicare claims for mammography ascertainment have been shown to be quite accurate [29], although no such validations have been made for the other breast modalities examined. Another limitation of using claims to identify imaging events is the lack of information regarding indication for exam, exam interpretation, and patient preferences. 

Our results, like those of other similar studies, characterize use of breast surveillance, but are not able to directly measure “appropriate” patterns of utilization. That is because, although guidelines exist for annual surveillance mammography, they are not based directly on performance/outcome data. At least one [30], but very few studies have examined the performance of annual mammography among breast cancer survivors, particularly in terms of false positives, recall rate, biopsy rate, and biopsy yield. Further, no empirical evidence currently informs how competing risks, such as comorbidities and influence surveillance guidelines for upper-ages. There is a similar lack of evidence for the efficacy and effectiveness of other imaging modalities and of other intervals, although the need for tailored surveillance approaches based on informed decision making, is documented [7]. Optimal regimens for surveillance breast imaging intervals, modalities, and targeted populations of breast cancer survivors are not known, but have tremendous population-wide implications for health, health care utilization, cost, and mortality.

## Figures and Tables

**Table 1:** Characteristics of 1219 women with a personal history of breast cancer in the New Hampshire Mammography Network (NHMN) and enrolled in Medicare (1998–2006).

Women's characteristics	*N* (%)
Age at diagnosis (years)*	
65–69	342 (28.1)
70–74	380 (31.2)
75–79	258 (21.2)
80+	239 (19.6)
Stage at diagnosis	
I	675 (55.4)
DCIS	233 (19.1)
IIA	209 (17.2)
IIB	65 (5.3)
III	37 (3.0)
Primary therapy^*£*^	
Mastectomy	832 (68.3)
Breast conserving surgery with radiation	187 (15.3)
Breast conserving surgery	117 (9.6)
Unknown/other	83 (6.8)
Family history of breast cancer	
No	875 (71.8)
Yes	337 (27.6)
Unknown	7 (0.6)
Comorbidities	
0	801 (65.7)
1	286 (23.5)
2+	132 (10.8)
Year of diagnosis	
1998	114 (9.4)
1999	189 (15.5)
2000	209 (17.2)
2001	176 (14.4)
2002	166 (13.6)
2003	186 (15.3)
2004	179 (14.7)
Education	
<High school	121 (9.9)
High school/ged	434 (35.6)
Some college	283 (23.2)
College or post-college graduate	273 (22.4)
Unknown	108 (8.9)
Race	
White	1210 (99.3)
Nonwhite	9 (0.7)

*Mean age in years (±SD) = 74 (±6); Median = 73 IQR: 69–77.

^*£*^Primary therapy given within 6 months after diagnosis.

**Table 2:** Women's characteristic and mammographic rates for each surveillance window.

	Surveillance windows			
	24 months	42 months	60 months	78 months
Numerator (*N*): number women who have an event	1088	774	518	296
Denominator (*N*): number of women in surveillance window	1219	895	619	363
Overall rate (%)	89.3%	86.5%	83.7%	81.5%

Characteristic	Rate %	Rate %	Rate %	Rate %

Age at diagnosis (years)				
65–69	88.0	86.5	87.1	86.0
70–74	92.9	91.2	86.5	85.4
75–79	89.2	87.2	86.2	81.0
80+	85.4	78.0	69.2	63.3
Stage at diagnosis				
I	91.3	88.7	86.2	84.5
DCIS	91.0	85.6	85.0	82.4
IIA	85.7	85.9	79.8	75.0
IIB	78.5	75.6	75.0	78.6
III	81.1	69.6	40.0	50.0
Primary therapy				
Mastectomy	90.6	85.9	82.6	79.3
Breast conserving surgery with radiation	97.3	97.7	97.8	95.4
Breast conserving surgery	88.0	88.1	79.2	88.9
Unknown/other	59.0	69.0	73.3	76.2
Family history of breast cancer				
No	88.7	86.2	82.6	80.3
Yes	90.8	87.2	86.5	84.9
Unknown	85.7	100.0	100.0	NA
Comorbidities				
0	87.8	87.9	84.8	81.3
1	91.6	84.5	81.5	79.4
2+	93.2	80.3	79.6	90.9
Education				
<High school	87.6	83.9	73.7	85.3
High school/GED	88.9	87.8	88.2	87.4
Some college	90.1	87.3	82.1	77.2
College+	89.4	88.6	87.1	83.3
Unknown	89.8	77.1	72.1	58.1

**Table 3:** Women's characteristics and ultrasound/MRI rates for each surveillance window.

	Surveillance windows			
	24 months	42 months	60 months	78 months
Numerator (*N*): number women who have an event	98	46	30	17
Denominator (*N*): number of women in surveillance window	1219	895	619	363
Overall rate (%)	8.0%	5.1%	4.8%	4.7%

Characteristic	Rate %	Rate %	Rate %	Rate %

Age at diagnosis (years)				
65–69	11.1	5.6	6.7	4.7
70–74	9.2	5.6	6.8	5.7
75–79	6.2	4.3	1.5	6.0
80+	5.4	6.4	3.9	6.1
Stage at diagnosis				
I	8.9	5.6	5.8	6.8
DCIS	7.3	6.3	5.3	4.4
IIA	7.7	4.0	1.8	4.4
IIB	7.7	6.7	12.5	0.0
III	10.8	4.4	0.0	0.0
Primary therapy				
Mastectomy	8.8	4.9	3.8	4.4
Breast conserving surgery with radiation	7.0	7.8	7.7	9.3
Breast conserving surgery	9.4	8.3	10.4	11.1
Unknown/other	6.0	2.8	6.7	4.8
Family history of breast cancer				
No	8.8	5.8	5.3	4.9
Yes	7.4	4.7	4.9	7.1
Unknown	0.0	0.0	0.0	NA
Comorbidities				
0	8.6	5.5	4.7	4.3
1	7.3	5.7	6.7	6.4
2+	9.1	4.9	5.6	18.2
Education				
<High school	8.3	4.6	1.8	8.8
High school/GED	6.7	5.3	5.7	4.4
Some college	9.9	5.9	6.4	6.3
College+	9.5	6.0	6.1	6.0
Unknown	8.3	4.8	1.6	3.2

**Table 4:** Women's characteristics and biopsy rates for each surveillance window.

	Surveillance windows			
	24 months	42 months	60 months	78 months
Numerator (*N*): number women who have an event	103	76	46	34
Denominator (*N*): number of women in surveillance window	1219	895	619	363
Overall rate (%)	8.4%	8.5%	7.4%	9.4%

Characteristic	Rate %	Rate %	Rate %	Rate %

Age at diagnosis (years)				
65–69	10.2	9.6	9.3	10.3
70–74	10.0	12.0	5.7	13.8
75–79	7.0	4.8	7.7	6.0
80+	5.0	5.2	6.7	2.0
Stage at diagnosis				
I	8.7	8.9	7.4	8.7
DCIS	7.3	5.8	8.9	11.8
IIA	6.2	7.4	4.6	8.8
IIB	15.4	20.0	8.3	7.1
III	10.8	4.4	20.0	16.7
Primary therapy				
Mastectomy	9.6	9.0	6.7	8.4
Breast conserving surgery with radiation	8.0	10.9	13.2	14.0
Breast conserving surgery	4.3	4.8	8.3	18.5
Unknown/other	3.6	4.2	3.3	4.8
Family history of breast cancer				
No	8.3	9.3	7.7	7.6
Yes	8.6	6.4	6.8	14.1
Unknown	14.3	0.0	0.0	NA
Comorbidities				
0	7.7	7.7	7.0	7.2
1	9.4	9.8	7.6	15.9
2+	10.6	11.1	11.1	18.2
Education				
<High school	6.6	8.1	5.3	14.7
High school/GED	8.8	8.2	7.0	7.4
Some college	8.8	7.8	10.7	12.7
College+	7.7	7.0	6.8	9.5
Unknown	10.2	15.7	4.9	3.2

**Table 5:** Multivariate poisson GEE models* for breast events.

	Mammogram			US/MRI			Biopsy			Number of breast events		
Characteristic	IRR	95% CI	*P*-value	IRR	95% CI	*P*-value	IRR	95% CI	*P*-value	IRR	95% CI	*P*-value
Age group												
65–69	1.00	(—, —)		1.00	(—, —)		1.00	(—, —)		1.00	(—, —)	
70–74	1.01	(0.97, 1.05)	0.76	0.98	(0.68, 1.40)	0.90	1.05	(0.80, 1.39)	0.72	0.98	(0.91, 1.05)	0.55
75–80	0.98	(0.93, 1.02)	0.31	0.65	(0.41, 1.02)	0.06	**0.66**	**(0.45, 0.96)**	**0.03**	**0.90**	**(0.83, 0.97)**	**0.01**
80+	**0.89**	**(0.83, 0.95)**	<**0.001**	0.73	(0.45, 1.20)	0.21	**0.54**	**(0.36, 0.83)**	**0.005**	**0.82**	**(0.75, 0.90)**	<**0.001**
Stage at diagnosis												
I	1.00	(—, —)		1.00	(—, —)		1.00	(—, —)		1.00	(—, —)	
DCIS	1.00	(0.95, 1.04)	0.92	0.97	(0.67, 1.42)	0.88	1.04	(0.76, 1.40)	0.82	1.00	(0.94, 1.07)	0.88
IIA	**0.93**	**(0.89, 0.99)**	**0.01**	**0.62**	**(0.40, 0.97)**	**0.04**	**0.66**	**(0.46, 0.94)**	**0.51**	**0.85**	**(0.79, 0.92)**	<**0.001**
IIB	**0.87**	**(0.79, 0.97)**	**0.01**	0.82	(0.44, 1.52)	0.52	1.15	(0.76, 1.73)	0.51	**0.81**	**(0.71, 0.94)**	**0.01**
III	0.86	(0.71, 1.01)	0.06	0.62	(0.24, 1.61)	0.32	0.73	(0.34, 1.59)	0.44	**0.65**	**(0.52, 0.81)**	<**0.001**
Primary therapy												
Mastectomy	1.00	(—, —)		1.00	(—, —)		1.00	(—, —)		1.00	(—, —)	
Breast conserving surgery with radiation	**1.09**	**(1.06, 1.12)**	<**0.001**	1.11	(0.76, 1.64)	0.59	1.12	(0.83, 1.51)	0.46	**1.22**	**(1.14, 1.30)**	<**0.001**
Breast conserving surgery	1.00	(0.94, 1.07)	0.90	**1.68**	**(1.06, 2.66)**	**0.03**	0.95	(0.59, 1.50)	0.82	**1.19**	**(1.08, 1.31)**	<**0.001**
Unknown/Other	**0.77**	**(0.68, 0.87)**	<**0.001**	1.07	(0.53, 2.18)	0.85	0.63	(0.34, 1.16)	0.14	**0.81**	**(0.70, 0.94)**	**0.01**
Family history												
No	1.00	(—, —)		1.00	(—, —)		1.00	(—, —)		1.00	(—, —)	
Yes	1.02	(0.98, 1.06)	0.22	0.88	(0.63, 1.23)	0.45	0.97	(0.75, 1.27)	0.86	1.03	(0.97, 1.09)	0.38
Unknown	0.96	(0.75, 1.24)	0.78	NA	NA	NA	0.78	(0.12, 4.92)	0.80	0.81	(0.63, 1.03)	0.09
Comorbidities												
0	1.00	(—, —)		1.00	(—, —)		1.00	(—, —)		1.00	(—, —)	
1	0.97	(0.93, 1.02)	0.24	0.93	(0.65, 1.34)	0.72	1.18	(0.88, 1.58)	0.27	0.97	(0.90, 1.05)	0.48
2+	0.97	(0.92, 1.03)	0.34	1.02	(0.64, 1.62)	0.95	1.09	(0.73, 1.62)	0.68	0.92	(0.84, 1.00)	0.06
Education												
Less than high school	1.00	(—, —)		1.00	(—, —)		1.00	(—, —)		1.00	(—, —)	
High school/ged	1.04	(0.97, 1.11)	0.24	0.87	(0.50, 1.52)	0.63	1.03	(0.67, 1.61)	0.86	1.06	(0.95, 1.17)	0.30
Some college	1.00	(0.93, 1.07)	0.95	1.02	(0.58, 1.82)	0.94	1.10	(0.69, 1.74)	0.69	1.00	(0.90, 1.12)	0.88
College or post graduate	1.02	(0.95, 1.10)	0.52	1.01	(0.57, 1.80)	0.96	0.97	(0.60, 1.55)	0.90	1.03	(0.92, 1.15)	0.60
Unknown	1.01	(0.93, 1.11)	0.74	0.91	(0.44, 1.89)	0.80	1.42	(0.85, 2.36)	0.18	1.02	(0.89, 1.16)	0.81

*Adjusted for all characteristics listed in the table and diagnosis year, number of primary or specialty care visits, and proportion of specialty care visits.

IRR: incidence rate ratio; 95% CI: 95% confidence interval.
